# Correction: Effects of Chinese medicine for COVID-19 rehabilitation: a multicenter observational study

**DOI:** 10.1186/s13020-022-00674-9

**Published:** 2022-10-24

**Authors:** Linda Li-Dan Zhong, Yi-Ping Wong, Chor-Yin Leung, Bo Peng, Zhi-Xiu Lin, Vivian Chi-Woon Wong Taam, Yi Luo, Hai-Yong Chen, Chao-Dong Chao, Chor-Fung Wong, Freddie Shung-Chi Tam, Kui Chan, Kwan-Yiu Lee, Lai-Fun Ho, Alan Yat-Lun Wong, Chi-Fung Choy, Bacon Fung-Leung Ng, Rowena How-Wan Wong, Yi-Bin Feng, Ching Liong, Zhao-Xiang Bian, COVID-19 CM Research Working Group

**Affiliations:** 1 School of Chinese Medicine, Hong Kong Baptist University, Hong Kong, China; 2 School of Chinese Medicine, The Chinese University of Hong Kong, Hong Kong, China; 3 School of Chinese Medicine, LKS Faculty of Medicine, The University of Hong Kong, Hong Kong, China; 4 United Christian Nethersole Community Health Service, The Chinese University of Hong Kong Chinese Medicine Clinic Cum Training and Research Centre (Tai Po District), Hong Kong, China; 5 HKFTU Workers’ Medical Clinics - Hong Kong Baptist University Chinese Medicine Clinic Cum Training and Research Centre (North District), Hong Kong, China; 6 Pok Oi Hospital - Hong Kong Baptist University Chinese Medicine Clinic Cum Training and Research Centre (Kowloon City District), Hong Kong, China; 7 The Hong Kong Tuberculosis Association, The University of Hong Kong Chinese Medicine Clinic Cum Training and Research Centre (Southern District), Hong Kong, China; 8 Pok Oi Hospital, The Chinese University of Hong Kong Chinese Medicine Clinic Cum Training and Research Centre (Yuen Long District), Hong Kong, China; 9 Pok Oi Hospital, The Chinese University of Hong Kong Chinese Medicine Clinic Cum Training and Research Centre (Shatin District), Hong Kong, China; 10 Haven of Hope, The Chinese University of Hong Kong Chinese Medicine Clinic Cum Training and Research Centre (Sai Kung District), Hong Kong, China; 11 Department of Medicine, Tseung Kwan O Hospital, Hospital Authority, Hong Kong, China; 12 Chinese Medicine Department, Hospital Authority, Hong Kong, China

## Correction: Chinese Medicine (2022) 17:99 https://doi.org/10.1186/s13020-022-00654-z

Following publication of the original article [1], the authors would like to make corrections after identifying the errors below which were caused by typo mistakes and the latest update of data analysis prior to publication but updating on some texts and Fig. 6A correspondingly had been missed. These changes will not impact the results nor alter the conclusion of this study.

**Correction (1): Clinical symptoms, diagnosis and health conditions during hospitalization**

Published version: Fever, fatigue, and dry cough were the most common symptoms, exhibiting in 59.3% (89 of 150), 55.3% (83 of 150), and 46.67% (69 of 150) participants, respectively.

Corrected version: Fever, fatigue, and dry cough were the most common symptoms, exhibiting in 59.3% (89 of 150), 55.3% (83 of 150), and 46.67% (**70** of 150) participants, respectively.

Reason for correction: it is a typo mistake made by the authors.

**Correction (2): Improvement in 6MWT performance**

Published version: However, with each increased age year, the distance achieve in 6MWT will decrease by 1.03 m.

Corrected version: However, with each increased age year, the distance achieve in 6MWT will decrease by **0.961** m.

Reason for correction: texts were not updated upon the latest update in data analysis with changes in linear mixed model equation. The authors believe it is necessary to correct the numerical values in text even though the change doesn’t impact on the findings. The corresponding data in Table 9 is correct and do not require any change.

**Correction (3): Lowered risk of COPD**

Published version: However, with each increased age year, the score obtained in LFQ will decrease by 0.11.

Corrected version: The statement “However, with each increased age year, the score obtained in LFQ will decrease by 0.11” has been removed.

Reason for correction: texts were not updated upon the latest update in data analysis with changes in linear mixed model equation. Although the statement would be correct under another calculation, it is no longer valid under the Model 2 calculation as published in the current article and serves no argument or discussion in this study. The authors believe that removing the statement can avoid inaccuracy and confusions.

**Correction (4): Fig. 6(A)**

Reason for correction: The authors identified an error in Fig. 6A which the graph was resulted from an earlier data analysis and was not from the latest one. The corresponding texts referring to this graph are correct and do not require any change.

The original article [1] has been corrected.

**Fig. 6:**
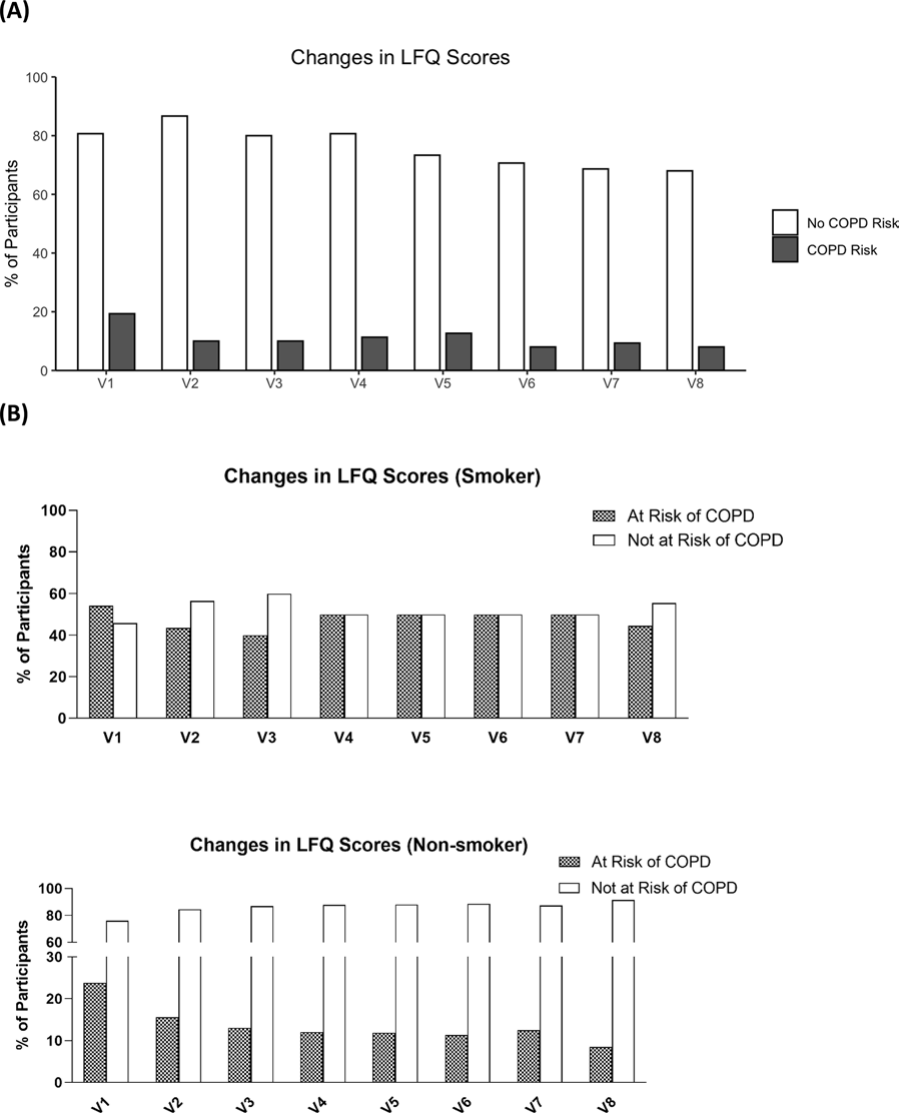
**A** Figure showed the percentage of participants with risk of COPD as accessed by their LFQ Scores. **B** Showed the percentage of non-smokers and smokers participants with risk of COPD
